# Geometrical Dependence on the Onset of Surface Plasmon Polaritons in THz Grid Metasurfaces

**DOI:** 10.1038/s41598-018-36648-x

**Published:** 2019-01-30

**Authors:** Gian Paolo Papari, Can Koral, Antonello Andreone

**Affiliations:** 1 Department of Physics, University of Naples “Federico II”, and CNR-SPIN, I-80125 Naples, Italy; 2 INFN Naples Unit, via Cinthia, I-80126 Naples, Italy

## Abstract

The transmission response of metallo-dielectric grid metasurfaces is experimentally investigated through Terahertz Time Domain Spectroscopy and the corresponding effective dielectric function is retrieved. Using a lumped element model we can determine the dependence of the effective plasma frequency (the transition frequency) on the metasurface filling factor F. The change of the transition frequency vs. F spans over one order of magnitude and sets the threshold between the metamaterial (homogeneous) and the photonic crystal (diffraction-like) regime, ruling the onset of two different Surface Plasmon Polaritons, spoof and high order. Field symmetry and spatial extension of such excitations are investigated for the possible applications of THz grid metasurfaces in bio- and chemical sensing and sub-wavelength imaging.

## Introduction

Surface Plasmon Polaritons (SPPs) are collective excitations induced at the boundary between metallic and dielectric materials. The underlying physics attracted a huge interest in the last few years because of their high potential in real world applications^1^. The possibility to control at will the activation energy, the extension, and the propagation length of SPPs make them optimal candidates for sub-wavelength imaging^1,2^, lithography^3^, surface sensing^4^, wave-guiding^5^, local field emission^6,7^, and many other applications.

Furthermore, the recent development of THz technology, based on reliable, compact, and high-performance sources and detectors^8^, is giving a boost to the study of tailored^9^ “cold” (in terms of energy) SPPs for the development of nearly zero refractive index metamaterials^10^ and for the control of enhanced transmission phenomena^11–17^.

The surface mode phenomenology may be divided into two categories: the *spoof* and the *tunneling* SPPs. Spoof Surface Plasmon Polaritons (SSPPs) refer to modes that are generated and remain confined at the metal-dielectric interface (MDI)^18^, with an appropriate electromagnetic perturbation having a frequency close to the plasma frequency *ω*_*p*_. The latter terminology pertains instead to high order modes (HOSPPs) excited at MDI but that propagate across the sample thickness, favoring the photon tunneling^15^.

In ordinary metals the plasma frequency can be as high as 10^15^ Hz^19^ and is given by ${\omega }_{p}=\sqrt{\,n\,{e}^{2}/{\varepsilon }_{0}{m}^{\ast }}$, where *n* is the charge carrier density, *e* is the elementary charge, *ε*_0_ the vacuum permittivity and m* the effective carrier mass. Spoof SPPs are upper bounded by the plasma frequency and their dispersion has the same signature of a standard SPP^20^. To excite them at lower frequencies, it is possible to decrease *ω*_*p*_ diluting the metal layer, with an operation of dimensional “squeezing” in which the unit cell volume *Vf* of a plane film is reduced to a value *V*_*u*_ < *V*_*f*_^20^. In this way, the diluted metal has an effective charge carrier density *n*′ = *nF*, where *F* = *V*_*u*_/*V*_*f*_ is the filling factor. Since the effective mass becomes related to *F*, *ω*_*p*_ of a diluted metal relies on the geometrical parameters of the unit cell only^21^.

**Figure 1:**
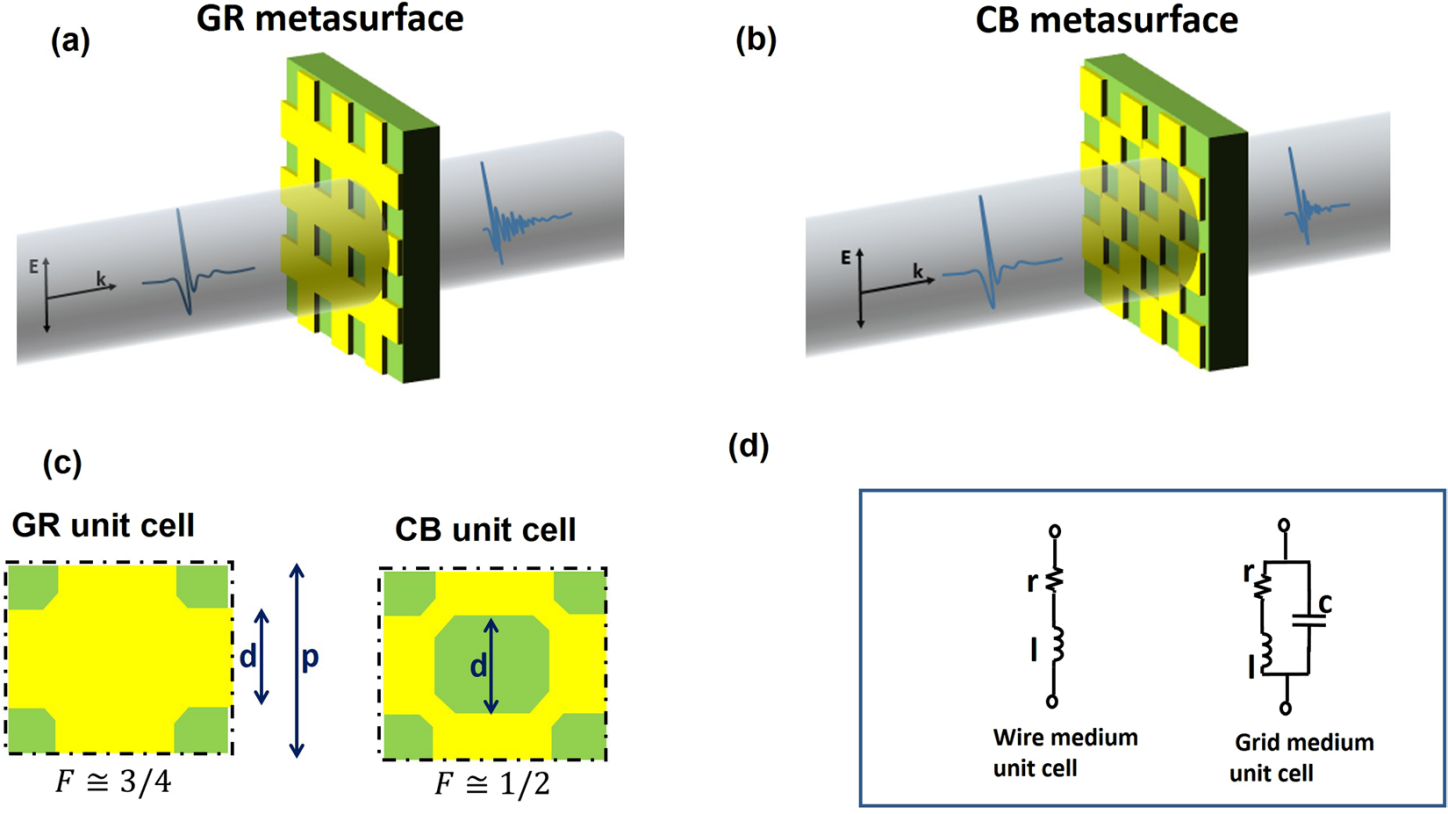
(**a**,**b**) pictorial representation (not in scale) of the THz beam impinging on the two grid metasurfaces. (**c**) Sketch of the GR and CB unit cell having same size d and periodicity p with the respective filling factor F. (**d**) Lumped element model for the unit cell of a wire and a grid medium.

We study the THz response of metasurfaces intended as diluted metals obtained through an appropriate combination of square symmetry conducting patches of side *d*, deposited over a dielectric substrate. Their periodical distribution over the surface composes a grid with periodicity *p* sized the unit cell (grid metasurfaces, GMs). In particular, we investigate the dielectric function of two GMs consisting of a patterned layer of copper, 30 μm thick, deposited over a FR4 foil with a nominal thickness *s* = 160 μm. Samples are realized through a standard process readily available from PCB industry. The conductive patches have the same periodicity *p* = 600 *μm* and the same size *d* ≈ 0.5*p*, composing a standard grid (GR, Fig. 1(a)) or a chessboard (CB, Fig. 1(b)) geometry. The latter grid medium can be obtained from the former one by removing the central metallic patch in the intersection of each perpendicular and parallel wire (Fig. 1(c)), resulting in a conducting network of wires.

To distinguish a GM from a standard MDI realized with an unpatterned metal layer, we define the reduced plasma frequency of the system as a *transition* frequency *ω*_*r*_, depending on the substrate permittivity and *F* only^22–24^.

We clearly show how *ω*_*r*_ provides the threshold for the onset of the high order SPPs^25^, typically observed in structured surfaces characterized by sub-wavelength holes^15^.

Time Domain Spectroscopy (TDS) is used to characterize in the transmission mode the response of the GMs and the onset of surface plasmon polaritons in the 0.1–1.0 THz range. Above *ω*_*r*_, tunnelling produced by the excitation of HOSPPs show up in the spectrum of both GR and CB samples as maxima in the amplitude. Experimental values of the transition frequency well agree with full wave electromagnetic simulations. Using a simple lumped element circuital model to describe the grid metasurfaces, we can obtain the analytical dependence of the transition frequency on *F*.

The analysis of the frequency spectrum allows to get a clear picture not only on the GM excited states, but also on the concept of dilution, somehow setting a threshold (ruled by *ω*_*r*_) between the metamaterial/homogeneous and the (metallic) photonic crystal regime. In this respect, we show that homogeneity can be seen as a frequency (energy) related condition rather than being determined from the trivial comparison between unit cell periodicity and the wavelength of the impinging signal. Furthermore, full wave electromagnetic simulations of the transmission response allow to identify the characteristics of the E-field at the HOSPP resonances, providing interesting information on the spatial symmetry and poles of the excitations in both grid metasurfaces.

## Results

**Figure 2:**
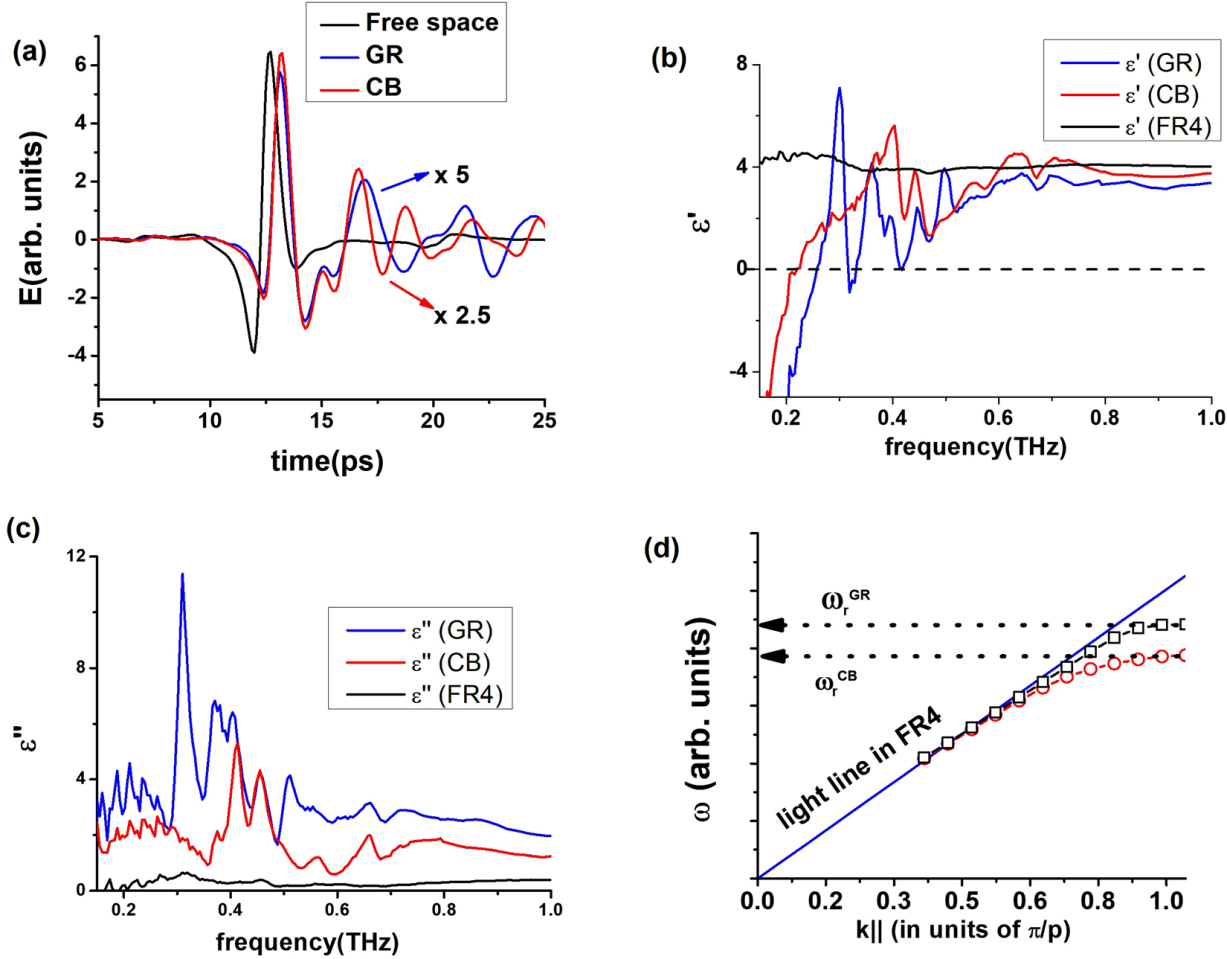
In (**a**) a detail of the acquired time dependent electric field transmitted through the free space (the reference, black line), and the CB (red line) and the GR (blue line) metasurfaces (magnified by a factor 2.5 and 5 respectively) is reported. (**b**) Real part ε’ of the dielectric function as a function of frequency: red, blue and black curves refer to the CB and GR media and the FR4 substrate respectively. (**c**) Same as in (**b**), but in terms of the imaginary part ε”. (**d**) Dispersion diagram of the GR (black square dots) and CB (red circle dots) metasurfaces varying the component of k|| along x direction. Arrows indicate the upper bounds given by the respective transition frequencies. The light line in the FR4 substrate (continuous blue curve) is also shown.

In Fig. 2(a), a detail of the acquired time dependent electric field (in arbitrary units) of the beam passing through the two metasurfaces under study is shown and compared with the free space. Black curve is the reference (air) signal *E*_*ref*_(*t*), whereas the blue and red curves refer to the transmission across GR, *E*_*GR*_(*t*), and CB, *E*_*CB*_(*t*), respectively. For the sake of clarity, *E*_*GR*_(*t*) and *E*_*CB*_(*t*) are shown scaled by the arbitrary factors reported in the figure. We assume that the THz signal is propagating along the z-direction, whereas the grid medium extends over the x-y plane.

The observed time shift in the peak maxima can be used to roughly evaluate the effective sample refractive index. Indeed, we estimate on average a delay of about *δt* ~ 0.5 *ps* (only marginally dependent on the geometry) which corresponds to a mean refractive index ${n}_{avg}\cong 1+\frac{c\delta t}{s} \sim 1.9$, lower than the corresponding value for FR4 (*n*_*FR4*_ ~ 2.1). This phenomenon can be accounted for by considering the dielectric function of the system, that in standard plasmonic structures^3^ is given by ${\tilde{\varepsilon }}_{eff}={\tilde{\varepsilon }}_{FR4}{\tilde{\varepsilon }}_{m}/({\tilde{\varepsilon }}_{FR4}+{\tilde{\varepsilon }}_{m}$). ${\tilde{\varepsilon }}_{FR4}\,$and ${\tilde{\varepsilon }}_{m}$ are the dielectric functions of the substrate and the metal layer respectively, the latter one being described through the standard Drude function ${\tilde{\varepsilon }}_{m}=1-{\omega }_{P}^{2}/{\omega }^{2}+i\omega {\omega }_{\tau }$ where *ω*_*r*_ is the relaxation frequency. Setting for a lossless diluted metal *ω*_τ_ = 0 and *ω*_*p*_ = 1 THz, we can easily verify that the effective refractive index of the composite metal-FR4 slab is lower than the bare FR4, in qualitative agreement with the experimental results.

Using a standard transmission function theory^8^, one can easily calculate the dielectric function $\tilde{\varepsilon }$ of each sample from the Fourier transform of the time signal. The grid metasurfaces do not present symmetry breaking in the plane of unit cells, therefore we use a retrieval process adopted in previous papers on homogeneous samples^26,27^. Since this procedure cannot account for the presence of diffracting/resonating processes above *ω*_*r*_, the dielectric function ${\tilde{\varepsilon }}_{GM}$ is obtained at its zero order. Results as a function of frequency are shown in terms of real *ε*_*GM*_′ (Fig. 2(b)) and imaginary *ε*_*GM*_″ (Fig. 2(c)) components. Red and blue curves in each graph refer to the behavior of ${\tilde{\varepsilon }}_{GM}$ in CB and GR samples respectively. The dielectric function of the bare FR4 substrate is also reported (black curve). All curves are affected by an uncertainty lower than about 7%, mostly related to the indetermination in the substrate thickness value.

Both the real and imaginary part of the dielectric response can be described dividing the frequency spectrum into three regions marked by the transition from the plasmonic regime and the onset of relevant diffraction process approximately at ${f}_{D}=c/p=0.5\,THz$. Full plasmonic behavior (*ε*_*GM*_′ < 0) can be clearly observed in Fig. 2(b) up to the transition frequency ${\omega }_{r,GR}/2\pi \approx 0.26\,THz$ and *ω*_*r*_,_*CB*_/2*π* ≈ 0.21 *THz* for GR and CB samples respectively.

Above the transition frequency the impinging radiation couples with higher order modes connected to the Bragg wave vectors (see eq. (12) in Methods), inducing HOSPP resonances in transmission that reflect in a dielectric function with dumped oscillations tending towards ${\tilde{\varepsilon }}_{FR4}$. For *f* > *f*_*D*_, diffraction starts smearing collective phenomena and further geometrical resonances are barely observed.

Following previous studies^28,29^, the resonating behavior of ${\tilde{\varepsilon }}_{GM}(\omega )$ can be in principle modeled through an electrical circuit whose impedance can fit the entire electrodynamics of the grid metasurfaces. However, an in-depth discussion on this is beyond the scope of our paper, focusing instead on the transition from SSPPs to HOSPPs.

The onset of SSPPs is testified by the dispersion diagram *ω*(k) of the fundamental mode^20^. This is reported in Fig. 2(d) presenting the standard monotonic growth upper bounded by the transition frequency, which scales according to the filling factor of the GM.

**Figure 3:**
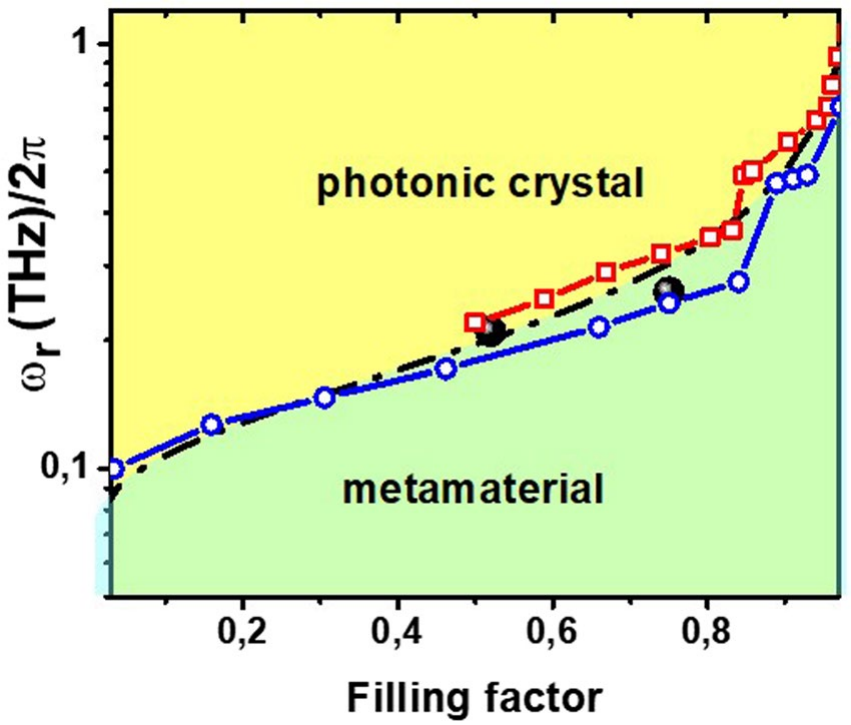
Plot of the transition frequency as a function of the filling factor. Red square and blue circle curves represent the results given by the full wave simulations for the CB and GR samples respectively. Black spheres represent the experimental values, ${{\omega }}_{{r},\text{CB}}/2{\pi }=0.21\,\text{THz}$ and ${{\omega }}_{{r},\text{GR}}/2{\pi }=0.26\,\text{THz}.$ The black dash-dotted curve is the best-fit using Eq. (3).

To evaluate the dependence of the transition frequency *ω*_*r*_ on *F*, we performed full wave electromagnetic simulations using commercial software. The filling factor can be related to the ratio η = *d/p*, yielding *F*_*GR*_ = 2*η* − *η*^2^ and *F*_*CB*_ = 2*η*^2^ − (2*η* − 1)^2^ for the GR and CB structure respectively (see Methods). Simulation results are shown in Fig. 3, as dot-dashed blue and red curves for the GR and the CB grid metasurfaces respectively. In the case of the CB sample the grid structure is lost for *F*_*CB*_ < 0.5, making the concept of plasma frequency meaningless. The function *ω*_*r*_(*F*) increases almost linearly as long as *F* < 0.8, to become exponential-like at higher values, so that its variation spans a frequency decade (from 0.1 to 1.1 THz).

Analytically, the *ω*_*r*_ dependence on *F* can be easily obtained using an approach based on lumped circuital elements^24^. The model calculates the grid medium dielectric function describing each unit cell as a RLC circuit, with parameters *r*, *l* and *c* representing the resistance, inductance and capacitance per unit length respectively. Specifically, while a simple wire medium can be described with a unit cell composed of a resistance in series with an inductance^23^, a grid metasurface can be modeled, at the lowest order, adding a capacitor in parallel to the basic RL unit cell (Fig. 1(d)). As shown in^23^ (and described in detail in Methods), the real part of the dielectric function for a generic grid medium can be expressed as

1 $${\varepsilon ^{\prime} }_{GM}={\varepsilon }_{\infty }+{\varepsilon }_{d}-\frac{{\omega ^{\prime} }_{p}^{2}{\tau }^{2}}{1+{\omega }^{2}{\tau }^{2}}$$

where ${\omega ^{\prime} }_{p}^{2}$ is the effective plasma frequency, *ε*_∞_ is the asymptotic permittivity, *ε*_*d*_ is the substrate dielectric constant and τ = *l/r* accounts for the relaxation time. The lumped element model defines the equivalence ${\omega ^{\prime} }_{p}^{2}=1/l\,p\,t$, where *t* is the medium effective thickness.

Following the results reported in^21^ one can express the geometry dependence of the lumped inductance as $l=\,\mathrm{ln}(\frac{1}{\sqrt{F}})/\pi$, so that

2 $${\omega ^{\prime} }_{p}^{2}=\frac{\pi \,{c}_{0}^{2}}{pt\,\mathrm{ln}(1/\sqrt{F})}$$

where *c*_0_ is the speed of light in vacuum.

By imposing the condition ${\varepsilon ^{\prime} }_{GM}=0$ and assuming a perfect conductor, from eq. (2) we can write the transition frequency as a function of *F*:

3 $${\omega }_{r}=\sqrt{\frac{\pi \,{c}_{0}^{2}}{pt\,({\varepsilon }_{\infty }+{\varepsilon }_{d})\,\mathrm{ln}(1/\sqrt{F})}}.$$

In the above formula, *t* represents the effective optical thickness of the GM taken equal to the dielectric substrate thickness (*t* = *s*).

Eq. (3) is plotted in Fig. 3 as a black dash-dotted curve. The analytical expression nearly overlaps the dependence given by the numerical data, showing that one can express the transition of a GM from the plasmonic regime in a very simple and general way. Black spheres represent the experimental values obtained from the real part of the dielectric function (see Fig. 2(b)), in very good agreement with both the analytical and numerical curves. The *ω*_*r*_(*f* ) curve sets also the transition from the “plasmonic” (homogeneous) to the”photonic” (diffractive) regime (see diagram in Fig. 3)). Above *ω*_*r*_ the GM assumes a dielectric behavior (${\varepsilon ^{\prime} }_{GM} > 0$), and because of the periodicity specific resonating modes are geometrically selected, enabling to consider the system as composed of separated metallic unit cells^30^.

**Figure 4:**
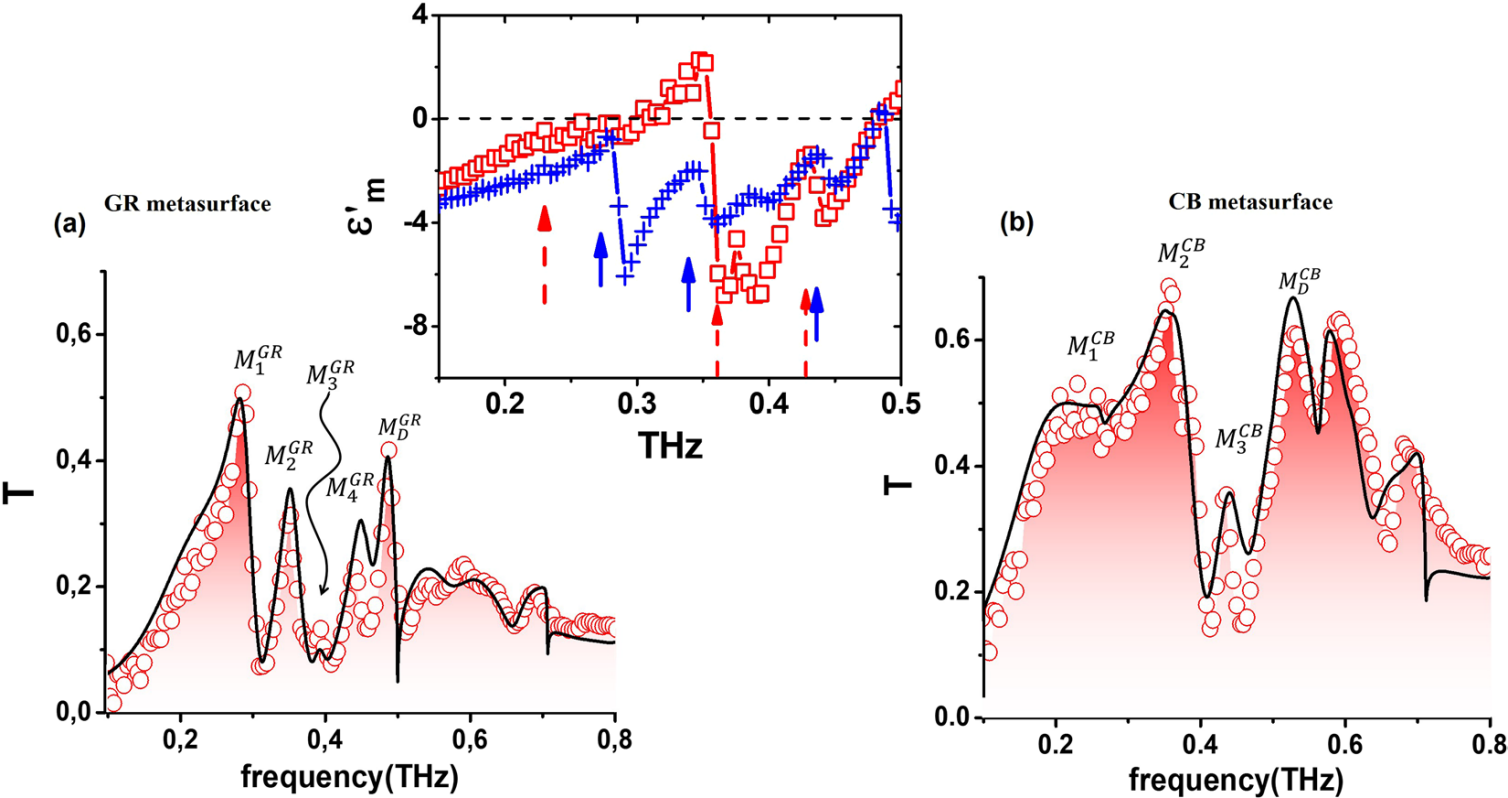
Experimental normalized transmission T measured (red open circles) through the GR (**a**) and CB (**b**) metasurface. Full wave simulations are reported as continuous black lines. Inset: real part of the dielectric function of the patterned metal layer only (see text). Red dashed and blue continuous arrows refer to the transmission peaks of the TM modes shown in (**a**,**b**) respectively.

Transmission peaks above *ω*_*r*_ are reported in Fig. 4(a,b). Black curves represent the full wave numerical simulations. In the graphs, HOSPP excitations are labeled as ${M}_{i}^{GR}$ and ${M}_{i}^{CB}$ respectively and are bounded at higher frequency by diffraction peaks labeled as ${M}_{D}^{GR,\,CB}$ lying very close to *f*_D_. Experimental results first display an almost monotonic and robust increase that ends in a first pronounced maximum (${M}_{2}^{CB}$ for the CB and ${M}_{1}^{GR}$ for the GR structure), followed by a deep and wide absorbance band. This “valley” is decorated with one (${M}_{3}^{CB}$) or two (${M}_{2}^{GR}$ and ${M}_{3}^{GR}$) peaks. ${M}_{4}^{CB}$ and ${M}_{5}^{GR}$ set the transition to the respective diffractive regime. Maxima are related to bright transverse magnetic (TM) modes occurring at quantized frequencies following the relation^14^ (see in Methods)

4 $${\nu }_{max}=\frac{c\,\sqrt{{m}_{x}^{2}+{m}_{y}^{2}}}{p\,{n}_{eff}^{{\max }}}$$

where ${n}_{eff}=\sqrt{{\varepsilon ^{\prime} }_{m}{\varepsilon }_{d}/{\varepsilon ^{\prime} }_{m}+{\varepsilon }_{d}}$ is the effective refractive index at the MDI. From eq. (4), it is possible to approximately estimate the value of *n*_*eff*_ using the frequency of transmission maxima in the SPP spectrum. In Tables 1 and 2 all states excited in the CB and GR samples respectively, and corresponding to the peaks marked in Fig. 4(a,b), are listed according to the triad $({n}_{eff}^{max},\,{m}_{x},\,{m}_{y})$. The effective refractive index ranges in between 2 and 3, in agreement with the corresponding values found above for ${\varepsilon ^{\prime} }_{GM}$ and reported in Fig. 2(b).

**Table 1:** Frequency, ${{n}}_{{eff}}^{{\max }}$, m_x_, m_y_ for the HOSSP modes on the GR metasurface.

Mode	Frequency (THz)	${{\boldsymbol{n}}}_{{\boldsymbol{eff}}}^{{\boldsymbol{\max }}}$	*m*_*x*_	*m*_*y*_
${{M}}_{1}^{{GR}}$	0.28	1.8	1	0
${{M}}_{2}^{{GR}}$	0.35	2.0	1	1
${{M}}_{3}^{{GR}}$	0.39	2.85	1	2
${{M}}_{4}^{{GR}}$	0.45	2.5	2	1

**Table 2:** Frequency, ${{n}}_{{eff}}^{{\max }}$, m_x_, m_y_ for the HOSSP modes on the CB metasurface.

Mode	Frequency (THz)	${{\boldsymbol{n}}}_{{\boldsymbol{eff}}}^{{\boldsymbol{\max }}}$	*m*_*x*_	*m*_*y*_
${{M}}_{1}^{{CB}}$	0.23	2.2	1	0
${{M}}_{2}^{{CB}}$	0.36	2.0	1	1
${{M}}_{3}^{{CB}}$	0.44	2.5	2	1

Although ${\varepsilon ^{\prime} }_{GM}$ assumes positive values in correspondence of the HOSPP band, the plasmonic features of the patterned metallic layer is preserved. Indeed, by employing the standard dielectric function of the metal-dielectric boundary ${\tilde{\varepsilon }}_{GM}={\tilde{\varepsilon }}_{m}\,{\tilde{\varepsilon }}_{FR4}/{\tilde{\varepsilon }}_{m}+{\tilde{\varepsilon }}_{FR4}$, we can extract ${\varepsilon ^{\prime} }_{m}$ using, in place of ${\tilde{\varepsilon }}_{FR4}$ and ${\tilde{\varepsilon }}_{GM}$, the function retrieved from the experimental data. The real part of the metal layer dielectric function is reported in the inset of Fig. 4, showing that the presence of HOSPPs is consistent with the condition ${\varepsilon ^{\prime} }_{m} < 0$. On the contrary, ${M}_{D}^{GR,\,CB}$ peaks correspond to null or positive values of ${\varepsilon ^{\prime} }_{m}$ and are therefore not accounted for as full SPP modes.

Minima in the GM excitation spectrum are related to modes grazing the metallic layer and are mostly due to the coupling with the FR4 substrate. The corresponding frequencies are very close to the precedent maxima^17,31^ having the same indices (*m*_*x*_, *m*_*y*_) and similar *n*_*eff*_.

At higher frequencies, Wood-Rayleigh (WR) anomalies arise at metal-air interface^32^ in correspondence of values ${\nu }_{min}=\frac{c\,\sqrt{{m}_{x}^{2}+{m}_{y}^{2}}}{p}\,({m}_{x}^{2}+{m}_{y}^{2}=1,2)$, as clearly seen as minima in the transmission response approximately at *f*_1,0_ = 0.5*THz*, *f*_1,1_ = 0.7*THz*.

Further information on SPP dynamics can be obtained by performing full wave simulations of the electric field spatial distribution of each excited state. In the following figures, the impinging radiation propagates along the z-axis with the electric field oriented parallel to the y-axis.

**Figure 5:**
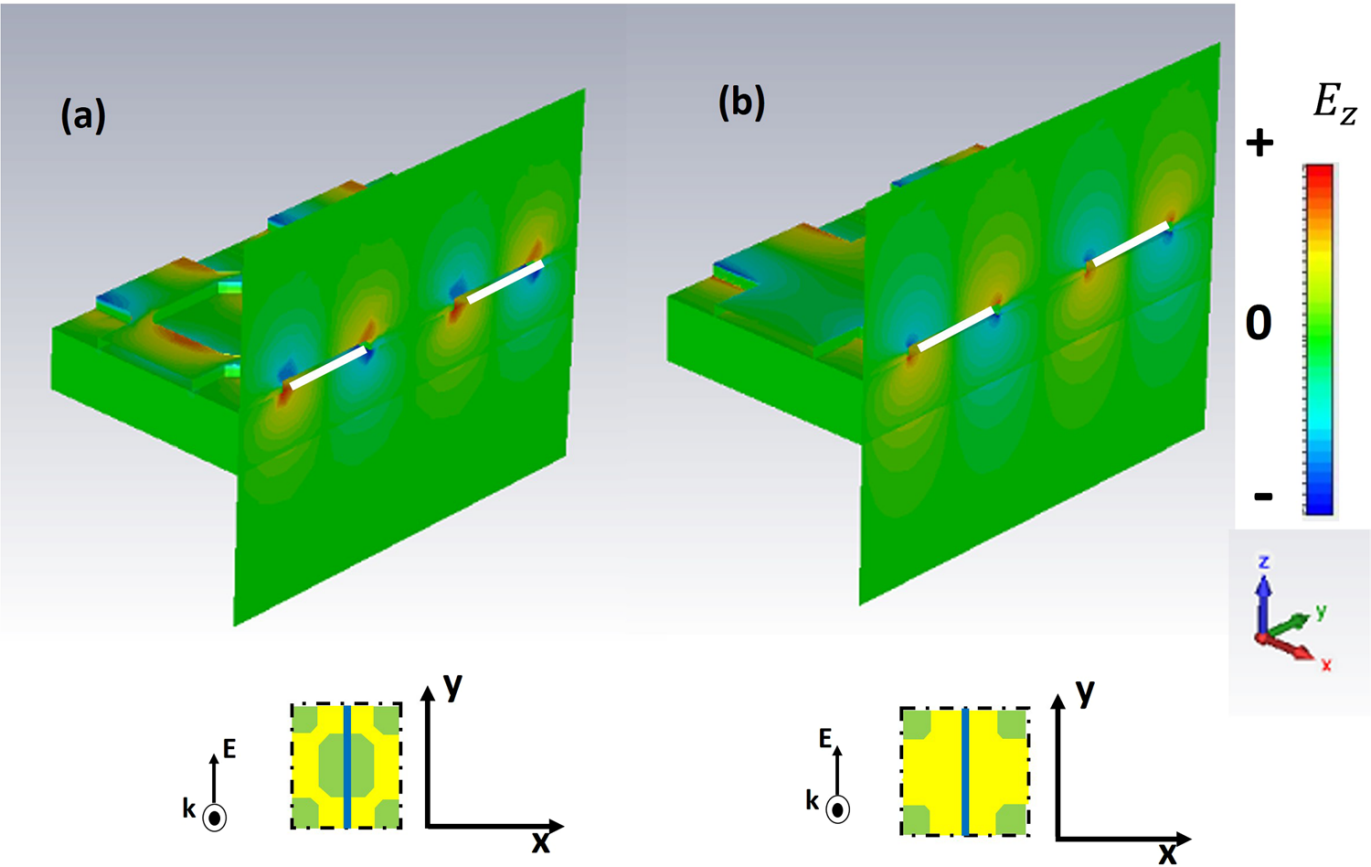
Simulated electric field intensity ${{E}}_{{z}}$ at metal/FR4 boundary for the CB (**a**) and the GR (**b**) metasurface. The dipolar distribution of SSPPs at f = 0.15 THz is projected along the (azure) cut reported in the sketch of each unit cell. The polarization of the impinging radiation is indicated as well.

We first report in Fig. 5 the field distribution of SSPPs for *ω* < *ω*_*r*_ (f = 0.15 THz) for the GR (a) and CB (b) metasurface. We choose the *E*_z_ component to highlight the dipolar symmetry of the field. In particular, the figure shows the electric field distribution in correspondence of the capacitive line where the GM displays a series of interrupted metallic patches.

For *ω* > *ω*_*r*_, high order SPPs come into play and show up in the excitation spectra of the GM samples. We have selected the three preeminent maxima in $|\tilde{T|}$. In particular, we refer to the peaks ${M}_{1}^{GR}$, ${M}_{2}^{GR}$,$\,{M}_{4}^{GR}$ for the GR and ${M}_{1}^{CB}$, ${M}_{2}^{CB}$, ${M}_{3}^{CB}$ for the CB metasurface.

**Figure 6:**
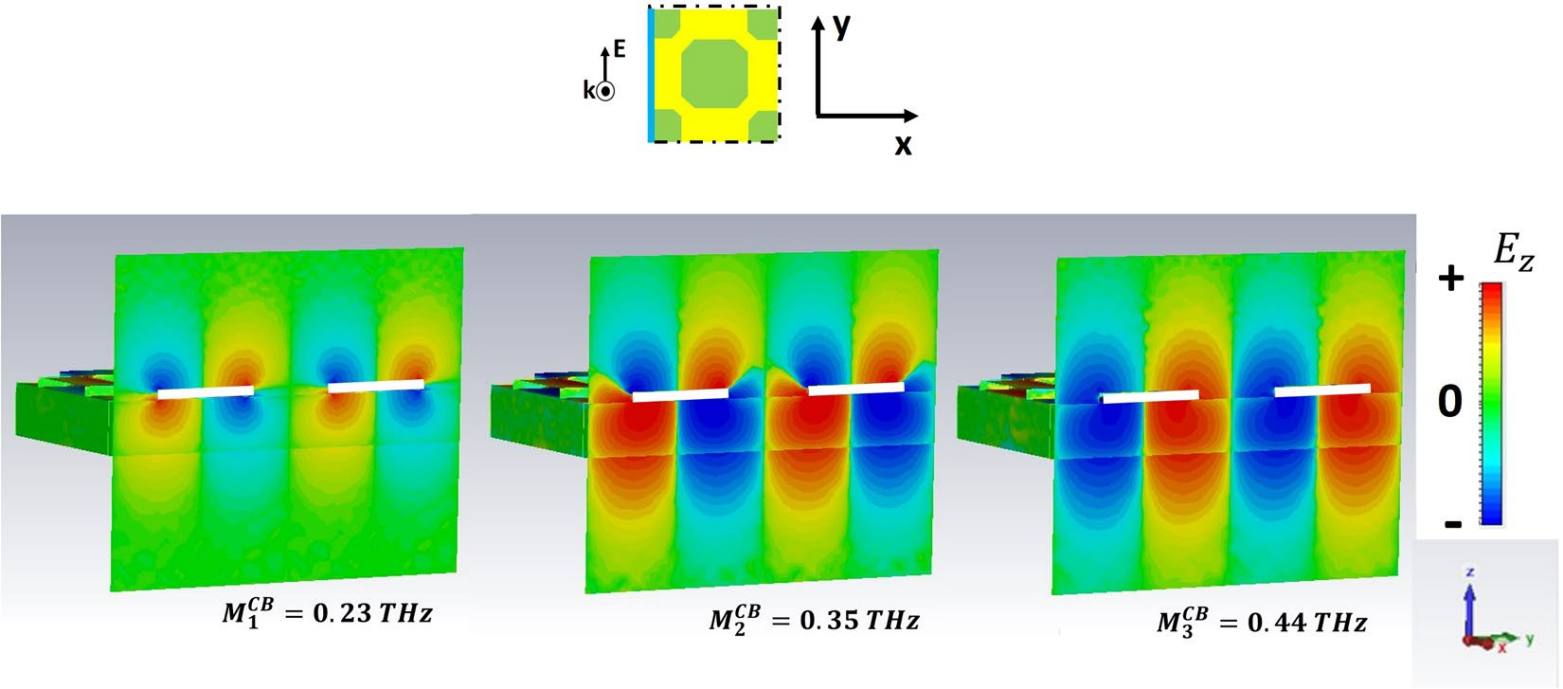
E vector field projected on the plane E-k of the impinging pulse for the CB metasurface. The azure line reported in the unit cell sketch describes the plane cut (capacitive line). The white rectangles represent the metallic parts.

**Figure 7:**
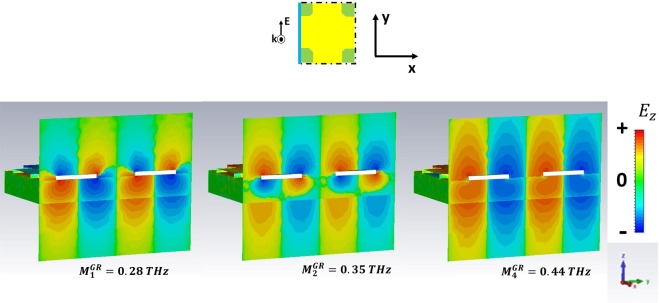
E vector field projected on the plane E-k of the impinging pulse for the GR metasurface. The azure line reported in the unit cell sketch describes the plane cut (capacitive line). The white rectangles represent the metallic parts.

**Figure 8:**
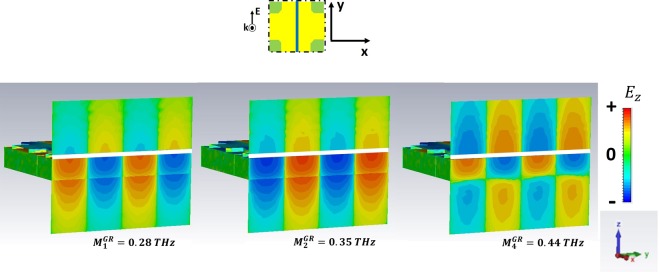
E vector field projected on the plane E-k of the impinging pulse, for the GR metasurface. The azure line reported in the unit cell sketch describes the plane cut (inductive line).

**Figure 9:**
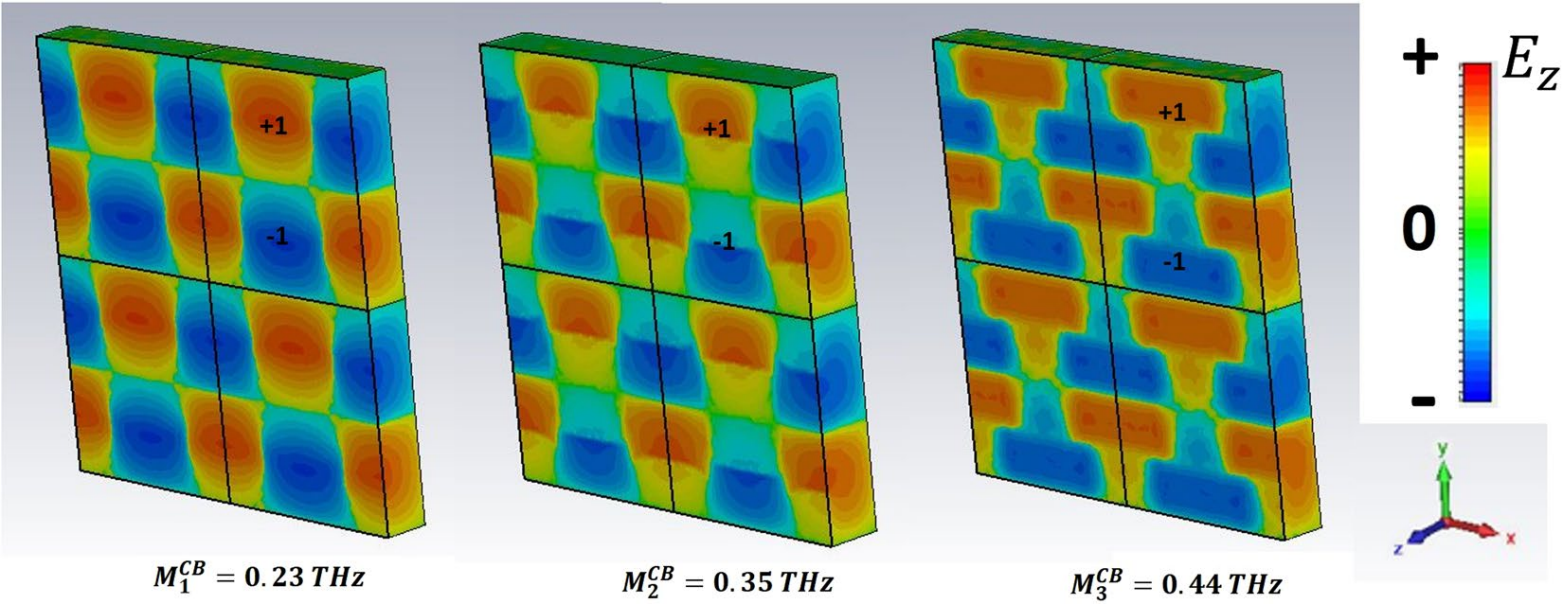
Electric field intensity at metal/FR4 boundary, in false color scale, for three different peaks of the CB metasurface. Dipolar symmetry is dominant at all reported resonant frequencies.

**Figure 10:**
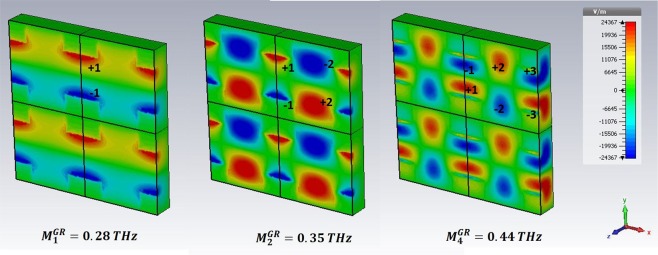
Electric field intensity at metal/FR4 boundary, in false color scale, for three different peaks of the GR rmetasurface. Numbers help to identify the poles of corresponding HOSPP modes. M_1_^GR^: 1, dipole; M_2_^GR^: 1, 2, quadrupole; M_4_^GR^: 1, 2, 3, hexapole.

The analysis of field distribution of HOSPPs is operated by choosing two different observation planes. In Figs 6, 7 and 8 the z-y plane is selected, so that one can look at the (mostly dipolar) excitations when they propagate along the sample thickness. Instead, in Figs 9 and 10 the HOSPP modes are studied in the x-y plane, to highlight the onset of multipolar features at the MDI.

In Figs 6 and 7 the *E*_z_ intensity along the capacitive line (see sketch in the figures) for the HOSPP modes in the GR and CB samples respectively is reported. ${M}_{1}^{GR}$, ${M}_{1}^{CB}$ and $\,{M}_{2}^{CB}$ modes present a dipole-like symmetry similar to the SSPP case, whereas the ${M}_{2}^{GR}$ excitation displays the onset of a further dipolar structure at the FR4/air boundary. Instead, the ${M}_{3}^{CB}$ and ${M}_{4}^{GR}$ present a dipolar distribution that extend all across the metasurface, due to the strong capacitive coupling of these modes caused by the condition $\,{\varepsilon ^{\prime} }_{GR,CB}\ge {\varepsilon ^{\prime} }_{FR4}$. Most of the dipolar field builds up inside the substrate underneath the metallic holes, producing field symmetry above and beyond the conducting layer.

In Fig. 8 the *E*_z_ intensity along the inductive line (see sketch in the figure) is reported for the GR metasurface. Here the electric field symmetry of HOSPPs differs from what shown along the capacitive line, mostly for the dipolar symmetries of ${M}_{2}^{GR}$,$\,{M}_{4}^{GR}$ which appear reversed.

The symmetries of dipoles distribution of HOSPP above and beyond the metallic layer directly depend on the different electromagnetic coupling at the sample interface (air and FR4 respectively).

Another interesting information regarding the field spatial distribution in correspondence of each HOSPP frequency is acquired by investigating *E*_z_ on the metasurface plane, as reported in Figs 9 and 10 for the CB and GR samples respectively. The former always displays a dipolar distribution independently of the mode order. On the contrary, the latter shows a multipolar expansion along the capacitive line as far as the frequency increases, so that we can easily identify a dipolar $({M}_{1}^{GR})$, a quadrupolar ${M}_{2}^{GR}$, and an hexapolar $({M}_{4}^{GR})$ character for the corresponding modes.

The onset of multipolar excitations in GMs corresponds to the behavior observed in metamaterials composed of separated unit cells^33–35^, confirming the observation of a diffraction-like response for *ω* > *ω*_*r*_.

## Conclusion

The THz response of grid metasurfaces realized using different patterns of a single square metal patch has been presented. These systems are valuable for investigating the whole family of SPP excitations observable below and above the transition frequency *ω*_*r*_, that separates the plasmonic/metamaterial region from the photonic crystal regime. *ω*_*r*_ sets the threshold between the energy necessary to excite either spoof SPPs, for *ω* < *ω*_*r*_, or high order SPPs, for *ω* > *ω*_*r*_. This transition frequency can be tuned and spans over one order of magnitude by only changing the filling factor of the GMs. Combining the experimental results and full wave electromagnetic simulations the dependence of the transition frequency on the metasurface filling factor *F* is retrieved. A simple lumped circuit is applied to model the GM unit cell, and from here an analytical expression of *ω*_*r*_(*F*) is obtained, depending on the geometrical characteristics of each grid and on the permittivity of the substrate only.

Since the fundamental SSPP mode behaves like in the case of an unpatterned MDI, *ω*_*r*_ represents the reference energy determining the upper bound for the homogeneous response of the system. Therefore, it can be used as a quantitative observable alternative to the wavelength threshold condition ${\lambda }_{thr}\,\sim \,p\sqrt{{\varepsilon }_{d}}$ below which the system discretizes.

The electric field at the cross section of the grid metasurfaces shows that the dipolar distribution of SSPPs is mostly localized at the metal/dielectric boundary, whereas HOSPPs basically present a spatial extension across the GM sample, being responsible for the electromagnetic tunneling and giving rise to enhanced transmission phenomena. Instead, the electric field at the MDI presents the onset of multipolar excitations in correspondence of transmission peaks.

The large field extension of HOSPPs makes grid metasurfaces simple and promising structures to perform either very accurate sensing experiments or near field sub-wavelength imaging.

## Methods

### Simulations

Full wave electromagnetic simulations of the samples under test are provided by employing a commercial software, CST Microwave Studio™, that basically solves Maxwell equations with finite difference methods^36^. Simulations are performed in the frequency domain and in unit cell configuration where the entire GM is assumed as composed of the periodical distribution of an elementary cell. A standard software library provides the copper electrodynamic parameters whereas the FR4 dielectric function is retrieved by direct measurements of the bare substrate.

### Measurements

**Figure 11:**
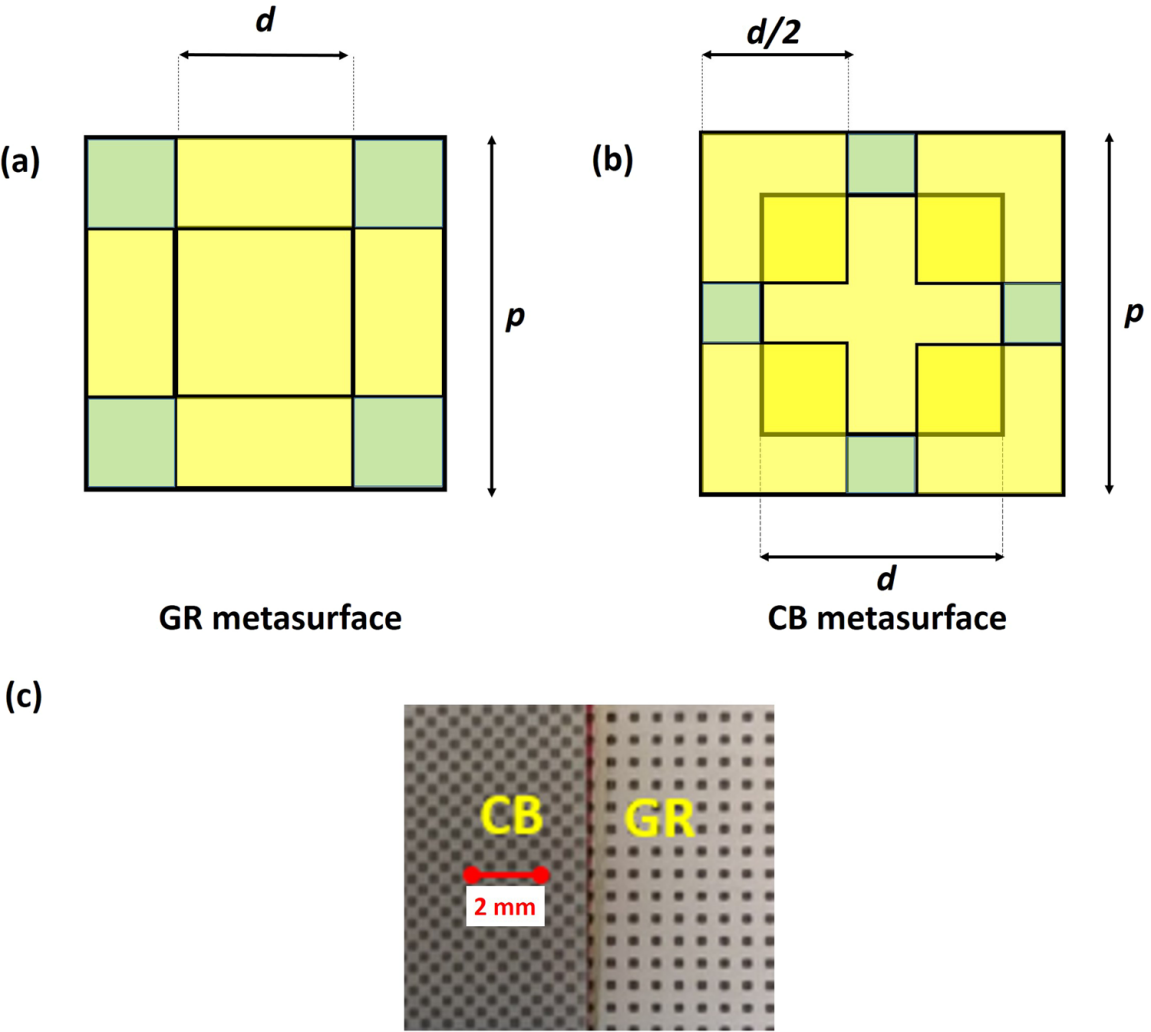
Sketch of the GR (**a**) and CB (**b**) unit cells for a generic size d of the metal patch. (**c**) A detail of the fabricated grid metasurfaces. In both samples the unit cell periodicity and the metallic patch side dimensions are 600 μm and 300 μm respectively.

Grid metasurfaces are printed over 10 cm × 10 cm FR4 foils using standard electrochemical and galvanic processes. Samples are cut in shape of squares, having an area of 2 × 2 cm^2^. In Fig. 11 a picture of the samples is shown.

The metasurfaces are placed on an aluminum plate with circular holes 15 mm in diameter. Similarly to previous experiments^26,27^, the sample holder can be finely moved in and out the THz beam to acquire in the same measurement session both the signal transmitted through the sample, *E*_*s*_(*t*), and the reference one in air, *E*_*air*_(*t*). In order to remove unwanted water absorptions, measurements are carried out in a dry nitrogen environment with humidity level of the order of 0.1% or less. Spectral analysis of samples are performed calculating the transmitted signal $\tilde{T}={\tilde{E}}_{s}/{\tilde{E}}_{air}$, where $\tilde{E}$ is the fast Fourier transform of the time signal.

### Electrical model of wire and grid media

The dielectric function of a bare metallic film, having thickness *t*, can be simply described sampling the film as composed by parallel array of wires (wire medium), whose electrical unit cell is constituted by a series of a resistance *R* and an inductance *L*, periodically distributed over a plane with step *p*. The total impedance of the structure matches with the impedance of the single unit cell ${\mathop{z}\limits^{ \sim }}_{u}=r+i\omega l$, where $r=R/p$ and $l=L/p$ (see Fig. 1(c)). Following the same approach described in^23^, the dielectric function ${\tilde{\varepsilon }}_{WM}$ of a wire medium can be obtained by using $\tilde{\varepsilon }=\,{\varepsilon }_{\infty }-i\tilde{\sigma }/\omega$ $({\varepsilon }_{0}=1)$ where the conductivity $\tilde{\sigma }$ is related to the unit cell impedance $\mathop{\sigma }\limits^{ \sim }=1/{\mathop{z}\limits^{ \sim }}_{u}\,pt$. After some algebra, the real and imaginary parts of ${\tilde{\varepsilon }}_{WM}\,\,$can be written in a Drude-like form as

5 $${\varepsilon ^{\prime} }_{WM}={\varepsilon }_{\infty }-\frac{{\omega ^{\prime} }_{p}^{2}{\tau }^{2}}{1+{\omega }^{2}{\tau }^{2}}$$

6 $${\varepsilon ^{\prime\prime} }_{WM}=\frac{{\omega ^{\prime} }_{p}^{2}\tau }{\omega (1+{\omega }^{2}{\tau }^{2})}$$

where ${\omega ^{\prime} }_{r}^{2}=1/l\,pt$ is the diluted plasma frequency, $\tau =l/r$ is the relaxation time, and ${\varepsilon }_{\infty }=1$ for standard metals. From eq. (5) it is easy to find the root frequency *ω*_*r*_ satisfying ${\varepsilon ^{\prime} }_{WM}({\omega }_{r})=0$:

7 $${\omega }_{r,WM}^{2}=\frac{{\omega ^{\prime} }_{P}^{2}}{{\varepsilon }_{\infty }}-\frac{1}{{\tau }^{2}}$$

Obviously, in a metal wire medium ${\omega }_{r}^{2}={\omega ^{\prime} }_{p}^{2}$ only for $\tau \to \infty$ $(r\to 0)$.

Extending previous approaches^23,24,37,38^, we qualitatively describe the transformation of a wire medium into a grid metasurface by introducing, in parallel to the previous unit cell, an effective capacitance *C* periodically distributed with step *p* (Fig. 1(c)). As a result, the new unit cell impedance is ${\mathop{z}\limits^{ \sim }}_{u}^{GM}={{z}^{WM}}_{u}\parallel 1/i\omega \,c$, where $c=C\cdot p$. From here, one can obtain the new dielectric function for a metal grid:

8 $$\begin{array}{c}{\varepsilon ^{\prime} }_{GM}={\varepsilon }_{\infty }-\frac{{\omega ^{\prime} }_{p}^{2}{\tau }^{2}}{1+{\omega }^{2}{\tau }^{2}}+\frac{{\omega ^{\prime} }_{p}^{2}}{{\omega }_{0}^{2}}\\ {\varepsilon ^{\prime\prime} }_{GM}={\varepsilon ^{\prime\prime} }_{WM}\end{array}$$

where ${\omega }_{0}^{2}=1/l\,c$. As expected, the capacitance modifies the reactive contribution of the material only, leaving unaltered the dissipative part. Looking for the new roots of ${\varepsilon ^{\prime} }_{GM}$ we obtain

9 $${\omega }_{r,WM}^{2}=\frac{{\omega ^{\prime} }_{p}^{2}}{{\varepsilon }_{\infty }+\frac{{\omega ^{\prime} }_{p}^{2}}{{\omega }_{0}^{2}}}-\frac{1}{{\tau }^{2}}$$

In other terms, the introduction of a capacitance per unit length lowers the metal-dielectric transition frequency, modifying the asymptotic value of permittivity to ${\varepsilon ^{\prime} }_{\infty }={\varepsilon }_{\infty }+{\omega ^{\prime} }_{p}^{2}/{\omega }_{0}^{2}={\varepsilon }_{\infty }+c/p\,t$. Since in both GMs the holes in the metal are square with side *d*, the capacitance can be assumed as composed by two armatures with area *t·(p-d)* and distance *(p-d)*, so that $C={\varepsilon }_{d}t$ and $c/pt=C/t={\varepsilon }_{d}$.

Following^21^, one can empirically extend the geometrical dependence of the lumped inductance given for a wire medium to the case of a grid (two-dimensional) structure, using the expression $l=\,\mathrm{ln}(1/\sqrt{F})/\pi$. In such a way, one can write the effective plasma frequency as

10 $${\omega ^{\prime} }_{p}^{2}=\frac{\pi \,{c}_{0}^{2}}{pt\,\mathrm{ln}(1/\sqrt{F})}$$

Inserting Equations (9) in (10), we achieve

11 $${\omega }_{r,GM}=\sqrt{\frac{\pi \,{c}_{0}^{2}}{pt\,({\varepsilon }_{\infty }+{\varepsilon }_{d})\,\mathrm{ln}(1/\sqrt{F})}}$$

where we assume that the metal behaves as a perfect conductor with ($r=0\,(\tau \to \infty )$). In other words, the transmitted radiation does not feel the metal dissipation because the conducting layer is thicker than the skin depth.

Following the schemes reported in Fig. 11(a,b), the filling factor *F* for each grid can be easily related to *η* = *d*/*p* yielding ${F}_{GR}=(2d\,p-{d}^{2})/{p}^{2}=\eta (2-\eta )$ and ${F}_{CB}=[2{d}^{2}-{(p-2d)}^{2}]/{p}^{2}=2{\eta }^{2}-{(2\eta -1)}^{2}$.

### SPP resonances

Resonance condition for each SPP implies that the *k*_*SPP*_ wave vector fulfills the Bragg constraints^17^

12 $${k}_{SPP}={k}_{0}+{m}_{x}{G}_{x}+{m}_{y}{G}_{y}$$

where ${k}_{0}=2\pi \,sin\vartheta /\lambda$ is the component of the impinging wave vector along the grid plane, *G*_*x*_ = *G*_*y*_ = 2*π*/*p* are the grating momentum wave vectors for a square array and *m*_*x*_, *m*_*y*_ are integers. Equation (12) accounts for a discrete set of wave vectors when the incoming photon perpendicularly hits the grid. This set is identified by the respective wavelengths which point at the maximum in transmission^17^

13 $${\lambda }_{max} \sim \frac{p}{\sqrt{{m}_{x}^{2}+{m}_{y}^{2}}}\sqrt{\frac{{\varepsilon ^{\prime} }_{m}{\varepsilon }_{d}}{{\varepsilon ^{\prime} }_{m}+{\varepsilon }_{d}}}$$

The minima are instead addressed as WR anomalies^17^^,^^32^, modes of diffracted light grazing the diluted metal surface. Accordingly, minima would not evoke SPPs and should roughly sit in correspondence of the following wavelengths^39^

14 $${\lambda }_{min}\cong p\sqrt{{\varepsilon }_{d}}/\sqrt{{m}_{x}^{2}+{m}_{y}^{2}}$$

where ${\varepsilon }_{d}$ can be either air or a dielectric substrate with *ε*_*d*_ > 1.

In the frequency domain, Eqs (12) and (13) can be re-written in terms of the effective refractive index of the coupling

15 $${\nu }_{max}\cong \frac{c\,\sqrt{{m}_{x}^{2}+{m}_{y}^{2}}}{p\,{n}_{eff}^{max}}$$

16 $${\nu }_{min}\cong \frac{c\,\sqrt{{m}_{x}^{2}+{m}_{y}^{2}}}{p\,{n}_{eff}^{min}}$$

where ${n}_{eff}^{min}$ and ${n}_{eff}^{max}$ depend on the different coupling of the metal grid with either the air or the dielectric substrate^17^.
